# Supplementary material for: Polypeptide Composition and Topology Affect Hydrogelation of Star-Shaped Poly(L-lysine)-Based Amphiphilic Copolypeptides
Source: Gels. 2021 Aug 30;7(3):131. doi: 10.3390/gels7030131 (PMC8482192; doi:10.3390/gels7030131)
Supplement: Supplementary file 1 [file gels-07-00131-s001.zip › gels-1337522-supplementary.pdf]

gels

MDPI

Article

Polypeptide Composition and Topology Affect Hydrogelation of Star-Shaped Poly(L-lysine)-Based Amphiphilic Copolypeptides

Thi Ha My Phan [1], Ching-Chia Huang [1], Yi-Jen Tsai [1], Jin-Jia Hu [3,*], and Jeng-Shiung Jan [1,2,*]

[1] Department of Chemical Engineering, National Cheng Kung University, Tainan 70101, Taiwan; myphan3008@gmail.com (T.H.M.P.); s110076@shsh.tw (C.-C.H.); iris870814@gmail.com (Y.-J.T.)
[2] Hierarchical Green-Energy Materials (Hi-GEM) Research Center, National Cheng Kung University, Tainan 70101, Taiwan
[3] Department of Mechanical Engineering, National Yang Ming Chiao Tung University, Hsinchu 30010, Taiwan
* Correspondence: jsjan@mail.ncku.edu.tw (J.-J.H.); jjhu@nctu.edu.tw (J.-S.J.)

Supplementary Materials

Table S1. Feed molar ratio, degree of polymerization (DP), number-average molecular weight (M_n) and molecular weight distribution (M_w/M_n) of *s*-PZLL homopolypeptides.

Polypeptide	Feed ratio	DP of ZLL	M_n [b]	M_w/M_n [b]
3*s*-PZLL$_{22}$	1:60	66	17000	1.37
6*s*-PZLL$_{21}$	1:120	126	35200	1.42

[a] Degree of polymerization (DP) of ZLL was calculated by ^{1}H NMR. [b] M_n and M_w/M_n were determined by GPC-LS.

Table S2. Feed ratios and block ratios between the ZLL block and the second block.

Polypeptide	Feed ratio	ZLL: Y[a]
3*s*-PZLL$_{22}$-*b*-PPhe$_{6.3}$	4 : 1	3.5 : 1
6*s*-PZLL$_{21}$-*b*-PPhe$_{5.3}$	4 : 1	4.0 : 1
3*s*-PZLL$_{22}$-*b*-PLeu$_{5.5}$	4 : 1	4.0 : 1
6*s*-PZLL$_{21}$-*b*-PLeu$_{4.4}$	4 : 1	4.8 : 1
3*s*-PZLL$_{22}$-*b*-PVal$_{5.1}$	4 : 1	4.3 : 1
6*s*-PZLL$_{21}$-*b*-PVal$_{5}$	4 : 1	4.2 : 1

[a] Block ratio of the ZLL block to the second block (Y block) was calculated by ^{1}H NMR.

Table S3. The corresponding d spacing obtained from SAXS profiles of 3-armed PLL-*b*-PY hydrogel samples.

Samples	Concentration (wt%)	d (Å)
**3*s*-PLL$_{22}$-*b*-PPhe$_{6.3}$**	5.0	273
	7.0	273
**3*s*-PLL$_{22}$-*b*-PLeu$_{5.5}$**	8.0	241.5
	9.0	241.5
**3*s*-PLL$_{22}$-*b*-PVal$_{5.1}$**	6.0	314
	8.0	314

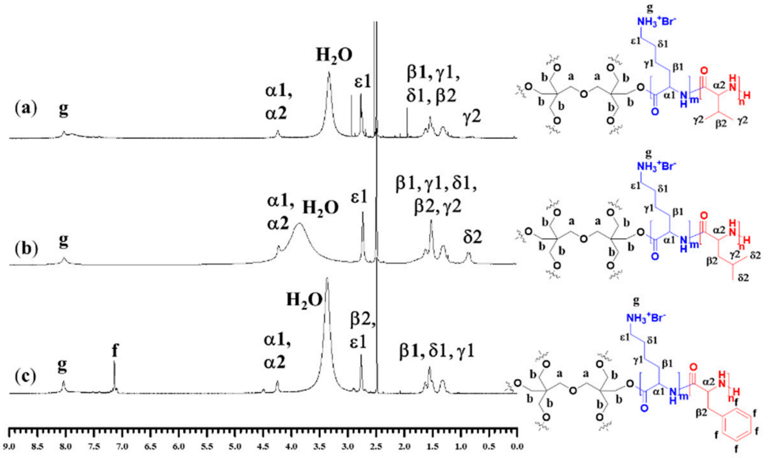

Figure S1. ^{1}H-NMR spectra of (**a**) 6*s*-PLL$_{21}$-*b*-PVal$_5$ in DMSO-d$_6$, (**b**) 6*s*-PLL$_{21}$-*b*-PLeu$_{4.4}$ in DMSO-d$_6$, and (**c**) 6*s*-PLL$_{21}$-*b*-PPhe$_{5.3}$ in DMSO-d$_6$.

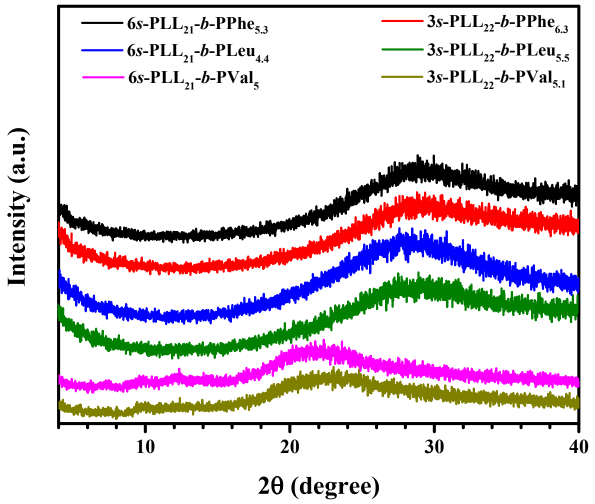

Figure S2. XRD patterns of freeze-dried 6s-PLL$_{21}$-b-PPhe$_{5.3}$ (5.0 wt%), 3s-PLL$_{22}$-b-PPhe$_{6.3}$ (5.0 wt%), 6s-PLL$_{21}$-b-PLeu$_{4.4}$ (5.0 wt%), 3s-PLL$_{22}$-b-PLeu$_{5.5}$ (8.0 wt%), 6s-PLL$_{21}$-b-PVal$_5$ (5.0 wt%), and 3s-PLL$_{22}$-b-PVal$_{5.1}$ (8.0 wt%) hydrogel samples.

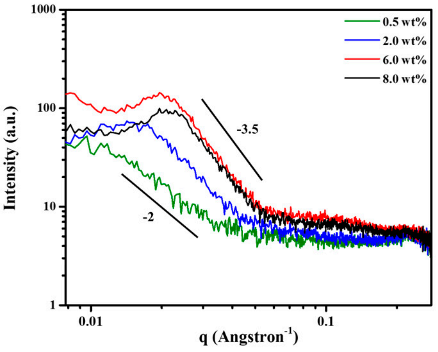

(**a**)

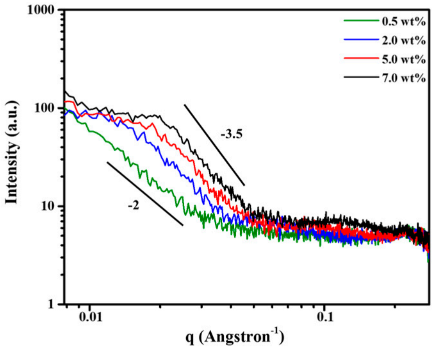

(**b**)

Figure S3. SAXS profiles of (**a**) 3*s*-PLL$_{22}$-*b*-PVal$_{5.1}$ and (**b**) 6*s*-PLL$_{21}$-*b*-PVal$_{5}$ sol and gel solutions at different polypeptide concentrations in DI water.
